# Supplementary material for: Effects of Mannan Oligosaccharides on Gas Emission, Protein and Energy Utilization, and Fasting Metabolism in Sheep
Source: Animals (Basel). 2019 Sep 28;9(10):741. doi: 10.3390/ani9100741 (PMC6826375; doi:10.3390/ani9100741)
Supplement: Supplementary file 1 [file animals-09-00741-s001.zip › animals-585569-SI.docx]

Supplemental Materials

Effects of mannan oligosaccharides on gas emission, protein and energy utilization, and fasting metabolism in sheep

Chen Zheng, Junjun Ma, Ting Liu, Bingdong Wei and Huaming, Yang

**Table 1.** Gas recovery rate by calibrating CO_2_ ^1.^

|  | Test 1 | Test 2 | Test 3 | Mean | CV^2^ (%) | |
| --- | --- | --- | --- | --- | --- | --- |
| Calibrating CO_2_ recovery rate (%) | | | | | |  |
| Chamber A | 96.5 | 96.2 | 96.5 | 96.40 | 0.147 | |
| Chamber B | 97.0 | 97.0 | 97.1 | 97.03 | 0.049 | |
| Chamber C | 97.2 | 97.4 | 96.9 | 97.17 | 0.211 | |
| Chamber D | 99.5 | 99.2 | 99.0 | 99.23 | 0.207 | |
| Chamber E | 101.2 | 101.5 | 101.4 | 101.37 | 0.123 | |
| Chamber F | 97.4 | 98.1 | 97.8 | 97.77 | 0.293 | |
| Chamber G | 98.2 | 98.8 | 98.6 | 98.53 | 0.253 | |
| Chamber H | 98.3 | 97.9 | 98.5 | 98.23 | 0.254 | |

^1^ Monitoring the CO_2_ concentration of each chamber until the concentration was stable. Then pumped calibrating CO_2_ 6.14 L into each chamber. After that, opened in-gas pump and off-gas pump and adjusted flow rate to 200 L / min. Then monitored the dynamic CO_2_ concentration of each chamber. When CO_2_ concentration is similar to the concentration of the first record, the test was over. And calculated the gas recovery rate by the following equation: Y = (x – a) × T × F / b. Where Y is recovery rate; x is average concentration; a is calibrating CO_2_ concentration; T is time for monitoring; F is gas flow; b is calibrating CO_2_ volume inserted into each chamber (6.14 L). The gas recovery should be range from 95 % to 105 %. ^2^ CV, coefficient of variation, = (standard deviation / mean) × 100%.
